# Advances in DNA Repair—Emerging Players in the Arena of Eukaryotic DNA Repair

**DOI:** 10.3390/ijms21113934

**Published:** 2020-05-30

**Authors:** Mateusz Kciuk, Karol Bukowski, Beata Marciniak, Renata Kontek

**Affiliations:** 1 Doctoral School of Exact and Natural Sciences, University of Lodz, Banacha Street 12/16, 90-237 Lodz, Poland; 2 Department of Molecular Biotechnology and Genetics, Faculty of Biology and Environmental Protection, University of Lodz, 12/16 Banacha St., 90-237 Lodz, Poland; karol.bukowski@unilodz.eu (K.B.); beata.marcniak@biol.uni.lodz.pl (B.M.); renata.kontek@biol.uni.lodz.pl (R.K.)

**Keywords:** DNA repair, sirtuin, circadian clock, long-noncoding RNA, heat shock protein

## Abstract

Genomic DNA is constantly damaged by factors produced during natural metabolic processes as well as agents coming from the external environment. Considering such a wide array of damaging agents, eukaryotic cells have evolved a DNA damage response (DRR) that opposes the influence of deleterious factors. Despite the broad knowledge regarding DNA damage and repair, new areas of research are emerging. New players in the field of DDR are constantly being discovered. The aim of this study is to review current knowledge regarding the roles of sirtuins, heat shock proteins, long-noncoding RNAs and the circadian clock in DDR and distinguish new agents that may have a prominent role in DNA damage response and repair.

## 1. Introduction

**Figure 1:**
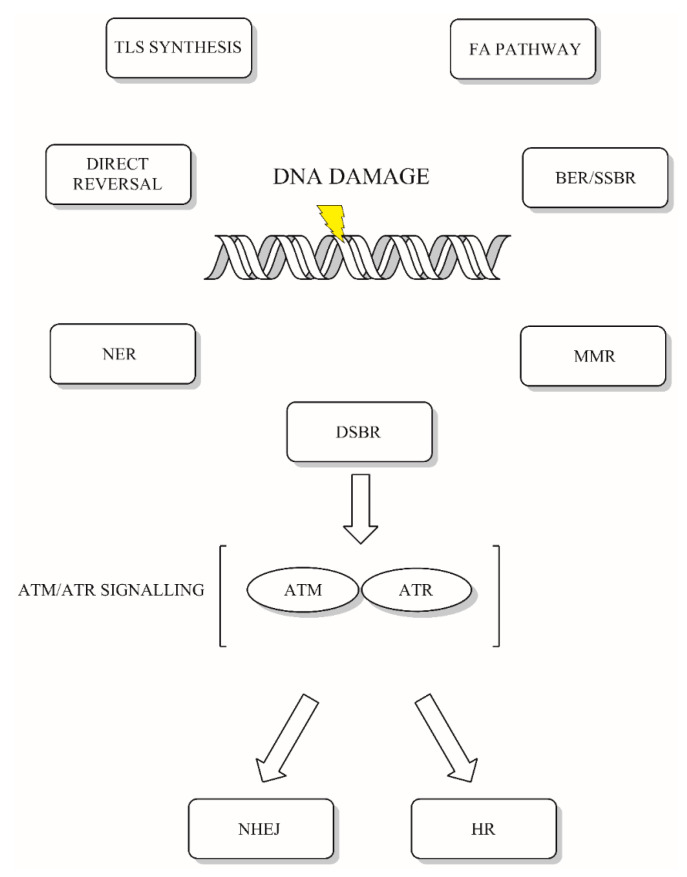
DNA damage response (DDR) in eukaryotes. Eukaryotic DNA response consists of systems of detection, signaling and repair of emerging DNA damage. The main DNA repair systems include direct reversal of damage, base excision repair (BER), nucleotide excision repair (NER), mismatch repair (MMR), Fanconi anemia pathway (FA), trans-lesion synthesis (TLS), single-strand break repair (SSBR) and double-strand break repair (DSBR): non-homologous end joining (NHEJ) and homologous recombination (HR). Double-strand breaks are signaled either by ataxia–telangiectasia and Rad3-related (ATR) or ataxia–telangiectasia mutated protein kinases (ATM) [2].

Eukaryotic DNA is constantly threatened by insults, either endogenous or exogenous in nature. Endogenous DNA damage results mainly from hydrolytic reactions with water and oxidative reactions with reactive oxygen species (ROS). In contrast, exogenous DNA damage arises from the activity of physical and chemical factors that damage DNA. These include exposure to UV light, ionizing radiation or alkylating agents. However, the examples presented above represent a small fraction of the actual range of DNA-damaging agents [1]. In response to such a wide array of deleterious factors, eukaryotic cells have evolved a DNA damage response system (DDR) that allows accurate repair of emerging damage (Figure 1) [2]. The nature of the damage determines the repair pathway choice, but most DNA repair systems work in a related manner. DDR consists of several key steps, that include damage sensing, signaling cascades and congruent damage repair. DDR has been previously discussed and reviewed elsewhere [1,2,3,4]. Here, we focus on new emerging trends in DNA repair research.

## 2. Sirtuins

Sirtuins (SIRT) represent a conserved family of proteins that regulate various intracellular processes, including glycolysis, gluconeogenesis, lipid metabolism and DNA repair [5]. Seven members of the mammalian sirtuin family (SIRT 1–7) have been identified so far. Sirtuins differ in cellular localization: SIRT6 and SIRT7 are nuclear proteins, while SIRT1 and SIRT2 are found both in the nucleus and cytoplasm. On the other hand, SIRT 3–5 perform their functions mainly in mitochondria [6,7].

Sirtuins are NAD+-dependent deacetylases that remove acetyl moieties form lysine residues of various proteins, including histones [8]. Moreover, sirtuins may act as mono-ADP-ribosyl transferases that conduct post-translational modification–mono-ADP-ribosylation of proteins [9]. The first identified sirtuin (Sir2) gene product was shown to regulate various processes, including gene silencing or DNA repair in *Sacharosmyces cerevisiae* [10,11,12,13]. Similarly to yeast Sir2, mammalian homolog SIRT1 was demonstrated to modulate DNA repair. In fact, SIRT1 displayed deacetylase activity towards multiple acetylated histone lysines, H4K16, H3K9, H3K56, H1K26 [14], H1K9 and H3K14 [15], affecting chromatin condensation status. For a long time, SIRT1 was regarded as a tumor promotor due to its elevated activity in some kinds of cancers [16]. However, it was later observed that the reduced activity of SIRT1 compromised genetic instability, and, thus, it was suggested that it may function as a tumor suppressor [17]. It was also demonstrated that SIRT1 regulated activity of TP53 via protein deacetylation on Lys320, Lys373 and Lys382. This interaction resulted in inhibition of apoptosis through diminished transactivatory potential of the protein in response to DNA damage [18,19]. SIRT1 plays a crucial role in double-strand break repair (DSBR) where it activates key components of the repair machinery, including Ku proteins, nibrin (NBS1) and Werner helicase (WRN) [20,21,22]. Moreover, cells defective in SIRT1 display diminished γH2AX (phosphorylated H2AX), breast cancer type 1 susceptibility protein (BRCA1), NBS1 and RAD51 foci formation following DNA damage. This results in impaired capability of damage repair in cells exposed to γ-radiation [17]. The possible consequences of this impaired ability to repair double-strand breaks (DSBs) comprise numerous translocations and chromosomal fusions [17,23]. Furthermore, effective recruitment of SIRT1 to damaged sites requires ataxia–telangiectasia mutated protein kinase (ATM) signaling and γH2AX foci formation [23]. Additionally, SIRT1 may be activated in CHK1-dependent phosphorylation on Thr530 and Thr540 residues [24]. Like SIRT1, SIRT6 also regulates gene expression through histone deacetylation. Among identified targets of SIRT6 are histone residues like H3K9 and H3K56. Histone deacetylation allows WRN helicase to associate with DNA and effectively serves the function of telomere structure maintenance [25,26]. In addition, SIRT6 recruits chromatin remodeling protein SNF2h to damaged sites supporting tumor suppressor p53-binding protein 1 (TP53BP1), replication protein A (RPA) and BRCA1 engagement in damage repair [27]. Similarly to SIRT1, SIRT6 depletion leads to genetic instability manifested by hypersensitivity to methyl-methanesulfonate (MMS) and ionizing radiation [25,26]. SIRT6 also plays a distinct role in non-homologous end joining (NHEJ) through interaction with DNA-dependent protein kinase catalytic subunit (DNA-PKcs) and Ku70/80 proteins, allowing their efficacious association with chromatin. SIRT6 can also deacetylate Lys539 and Lys543 of Ku70 protein and stimulate its activity [25,28]. SIRT6 is involved in base excision repair (BER), where it mono-ADP ribosylates poly(ADP-ribose) polymerase (PARP1) and stimulates its enzymatic activity. This leads to more effective signaling of single-strand breaks (SSBs) and facilitates access of X-ray repair cross-complementing protein 1 (XRCC1) and pol β to the site of damage during repair [29,30]. Moreover, SIRT6 was shown to stimulate MYH glycosylase and apurinic/apyrimidinic endonuclease (APE1) during BER [31]. Furthermore, SIRT1 can take part in BER through deacetylation of the aforementioned APE1 endonuclease and thymine DNA glycosylase (TDG) [32,33]. Besides BER, SIRT1 has been shown to be implicated in stimulation of nucleotide excision repair (NER) via deacetylation of Lys63 and Lys67 of xeroderma pigmentosum proteins XPA [34] and XPC [35]. Recently, Jung et al. reported that SIRT1 could modulate expression of two key proteins involved in DNA mismatch repair (MMR), mutS homologs (MSH2 and MSH3). Furthermore, they demonstrated that SIRT1 inhibition triggered apoptosis of embryonic stem cells due to increased genomic instability [36].

**Figure 2:**
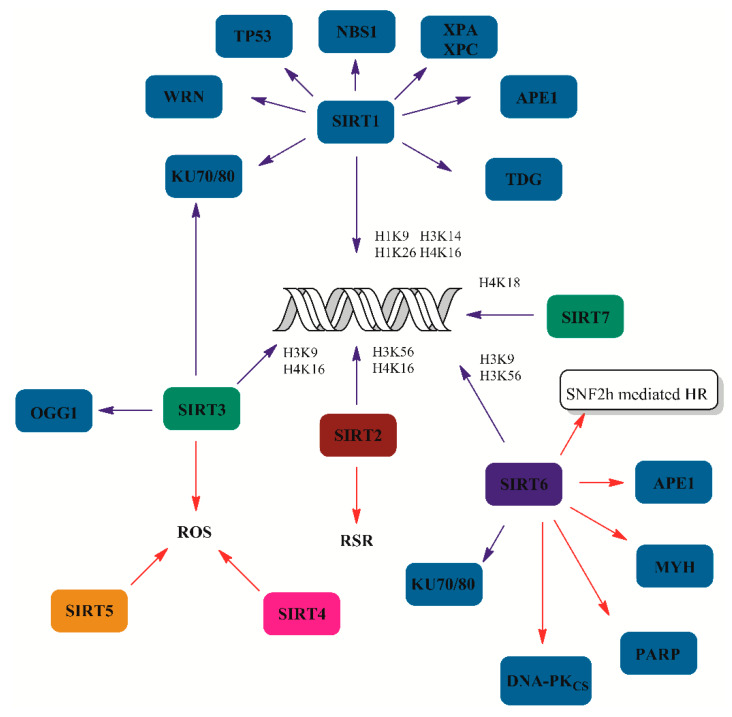
Role of sirtuins (SIRTS) in DNA repair. Blue lines indicate deacetylation reactions. Red lines represent other interactions between sirtuins and DNA damage response components. Most sirtuins, excluding SIRT4 and SIRT5, possess deacetylase activity toward multiple acetylated lysine residues of histone proteins. SIRT1 and SIRT6 have a wide range of substrates including BER (DNA glycosylases MYH and TDG; AP endonuclease APE1) and NER components (xeroderma pigmentosum proteins XPA and XPC), DSB proteins including KU protein, nibrin (NBS1), DNA-dependent protein kinase, catalytic subunits (DNA-PKcs), PARP1, and other DDR-related factors such as WRN and TP53 protein. Mitochondrial sirtuins (SIRT3, SIRT4 and SIRT5) prevent ROS-induced DNA damage in mitochondria. SIRT3 deacetylates and stimulates the activity of OGG1 glycosylase.

In contrast, other members of the sirtuin family have not been examined so thoroughly. SIRT2 was implicated as involved in DNA replicative stress response (RSR). The main identified protein target of SIRT2 remains cyclin-dependent kinase (CDK9). Like other previously mentioned sirtuins, SIRT2 can deacetylate histone proteins like H4K16 and H3K56 [37,38]. SIRT7 has not been well studied either. The main identified substrates of SIRT7 are histone proteins, primarily H3K18 [39,40]. In mouse models, SIRT7 knockout led to increased acetylated H3K18 level, which contributed to reduced DSBR through the NHEJ pathway [41]. Due to mitochondrial localization, other sirtuins may not play a direct role in nuclear DNA repair. However, they may affect crosstalk between mitochondrial and nuclear DNA concerning DNA repair. DDR proteins are important constituents of such signaling events and they may represent a potential pool of sirtuin targets. However, the role of SIRT3, SIRT4, and SIRT5 in DNA repair remains to be elucidated [42]. Mitochondrial sirtuins are responsible for maintenance of genetic stability of mitochondrial DNA, mainly through ROS scavenging. For example, SIRT3 was demonstrated to regulate glutathione-dependent redox balance in mitochondria [43]. In addition, SIRT3 works as a deacetylating enzyme with a preference towards H3K9 and H4K16 or Ku70 proteins [44,45]. Another well-established substrate of SIRT3, OGG1, is a member of DNA glycosylases involved in BER. SIRT3 can deacetylate OGG1 glycosylase and stimulate its activity. This seems to be crucial regarding the amount and detrimental consequences of 8-oxoguanine formation in DNA [46,47]. SIRT4, on the other hand, arrests cell cycle progression in response to DNA damage, providing more time for DNA repair, and thus delaying apoptosis [48]. SIRT5 possesses multiple enzymatic activities but little is known considering its role in DNA repair [49]. The role of sirtuins in DNA repair has been summarized in Figure 2. 

Sirtuins constitute an interesting family of specialized enzymes that regulate various aspects of DNA repair. They work both as protein activators and chromatin-structure-modifying enzymes. Deacetylation carried by sirtuins represents a basic epigenetic mechanism. Histone modifications including deacetylation and poly-(ADP)-ribosylation compromise an essential part of physiological ageing processes that are involved in the pathogenesis of ageing-related diseases, including cancer. Sirtuins may exhibit both suppressing and cancer-promoting activities; thus, understanding of underlying sirtuin-dependent tumorigenic mechanisms can lead to development of new antineoplastic therapies. Sophisticated crosstalk between sirtuins and DNA repair proteins represents an unknown area of research. From an evolutionary standpoint, it is still unclear why sirtuins evolved as a group of proteins that regulate such a wide array of processes. Moreover, the activity of sirtuins can be reshaped on different levels. Sirtuins, like other proteins, may undergo post-translation modifications that affect their catalytic activity [24,50,51,52]. MicroRNAs can influence SIRT mRNA stability and thus decrease SIRT levels to certain extent. This further affects complex sirtuin-dependent regulatory networks [53].

## 3. Long Non-coding RNAs

Long noncoding RNAs (lncRNAs) comprise an abundant group of diverse RNA molecules with length exceeding 200 nucleotides [54]. These non-coding RNAs perform different biological functions, including transcription regulation, modulation of chromatin structure through DNA methylation, histone modification and chromatin remodeling, posttranscriptional regulation, modulation of protein activity, and others extensively reviewed elsewhere [55,56]. The function of lncRNAs is highly dependent on their subcellular localization. There are three different fractions of lncRNAs reckoning their place of action: cis nuclear lncRNAs that are localised close to their sites of transcription, lncRNAs that perform functions in the nucleus but regulate expression of genes distant from their own sites of transcription (in a trans-dependent manner) and lncRNAs that need to be exported (transported) to cytoplasm to perform their regulatory functions [54]. Furthermore, based on their immediacy to protein coding genes, lncRNAs have been classified into several groups: sense, antisense, intronic, intergenic transcripts and pseudogenes.

Significant scientific progress has been made regarding the role of lncRNAs in DNA repair. LncRNAs are considered to play a prominent role in DSB repair. They have been shown to alter DSB repair through several mechanisms: (a) through TP53 activity modulation at transcriptional and translational level, (b) through recruitment of chromatin remodelers that modulate the access of DNA repair proteins to the site of damage, (c) by working as scaffolds and mediators for DNA repair proteins, and (d), last but not least, acting as sponges for various DNA-damage-associated miRNAs [57]. 

As previously mentioned, DSBs lead to recruitment of DNA damage sensors, such as MRN complexes and Ku proteins, at the site of DNA damage. This is followed by firing of signaling cascades and downstream protein activation [58]. The key component activated upon DSB is ATM protein kinase. ATM phosphorylates H2AX histones at the site of damage, leading to γH2AX foci formation at break sites [59]. Moreover, ATM activation leads to CHK1- and CHK2-dependent TP53 phosphorylation [2]. TP53, often perceived as a “guardian of the genome”, is one of the best-studied tumor suppressor proteins. It has been estimated that almost half of human tumors carry a mutation in the *TP53* gene. Activation of TP53 upon DNA damage leads to either cell cycle arrest or apoptosis depending on the nature and severity of the damage. TP53 acts as a key transcriptional regulator of different proteins inside the cell [60]. Moreover, CHK1/2 activation leads to inhibition of cyclin-dependent kinase activity that slows down or arrests the cell cycle in G1-S or G2-M phase [61]. The expression of lncRNAs can be induced following DNA damage. This may occur in a TP53-dependent manner. Additionally, some lncRNAs may regulate expression of TP53 downstream targets, further complicating the interactions.

The examples of *TP53*-linked lncRNAs are *lincRNA-p21* [62] and *PANDA* [63], both located upstream of *CDKN1A (p21)* gene. P21 is a protein that binds to certain CDKs, forming inactive complexes that compromise cell cycle arrest and apoptosis. *lincRNA-p21* was shown to repress transcription induced by TP53 through interaction with heterogeneous nuclear ribonucleoprotein-K (hnRNP-K), which constitutes an important component of repressor complexes. These complexes are recruited to the promoters of downstream *TP53* transcriptional targets and prevent effective *TP53*-mediated transcription [62]. In contrast, *CDKN1A* upstream lncRNA, *DINO*, was shown to stabilize TP53 protein and stimulate its transactivatory activity [64]. Other lncRNAs, like *WRAP3α* lncRNA directly bind to *TP53* mRNA after DNA damage to stabilize the protein, and thus affect its level inside the cell [65]. *LINP1*, on the other hand, works as a scaffold for NHEJ proteins (Ku70–Ku80 and DNA-PKcs) during DNA repair, where it promotes the religation of broken DNA strand ends [66]. Another lncRNA worth mentioning, *MALAT1*, constitutes a link between sirtuins and *TP53*. *MALAT1* sequesters DBC1, a negative regulator of SIRT1, and thus promotes SITR1-mediated deacetylation of TP53. This results in altered expression of TP53 target genes and *TP53*-linked lncRNAs [67,68,69]. Misteli et al. demonstrated that intergenic lncRNA *DDSR1* expression could be elevated in response to DNA-damaging drugs. *DDSR1* induction is greatly dependent on ATM and NF-Κb activation but TP53 is not necessary for its induction—nevertheless, it still may regulate its expression. Interestingly, *DDSR1* can regulate TP53-target gene expression. Moreover, *DDSR1* knockdown leads to impaired homologous recombination (HR) and upregulation of TP53-dependent gene expression, especially of those genes that contribute to cell proliferation [70,71]. The choice between HR and NHEJ repair pathways is further attributed to two noncoding RNAs—*CUPID1* and *CUPID2*—located in the enhancer region of the *CCND1* gene, coding for cyclin D1 [72]. The lncRNA *GUARDIN* plays an important role in genome stability maintenance. Sequestering of miRNA-23a by *GUARDIN* leads to sustained expression of telomeric repeat factor 2 (TRF-2), which prevents chromosome end fusion. Furthermore, *GUARDIN* regulates the stability of BRCA1 and promotes its association with BRCA1-associated RING domain protein (BARD1) for effective HR [73]. *TODRA*, an antisense lncRNA transcribed upstream of the RAD51 recombinase gene, has also been shown to be implicated in HR, where it regulates RAD51 expression and protein activity [74]. Numerous lncRNAs have been confirmed to play a role in DDR. These include the following lncRNAs: *ANRIL* [55], *BARD1 9´L* [75], *Gadd7* [76,77], *HOTAIR* [78,79] *JADE* [80], *LincROR* [81], *LIRRE* [82], *MDC1-AS* [83], *NEAT1* [84], *PCAT-1* [85,86,87], *PINCR* [88], *PINT* [89,90], *PURPL* [91], *PR-lncRNA-1*, *PR-lncRNA-10* [92], *TERRA* [93,94].

The importance of lncRNAs in cellular physiology is certainly unquestionable. LncRNAs play a significant role in DNA repair through various cis and trans mechanisms. Besides the influence of lncRNAs in gene expression, they can act as scaffolds for DNA repair proteins or work as miRNA scavengers, affecting both the activity and abundance of DDR components. It remains unclear how the primary and secondary structure of lncRNAs molecules affects DDR protein activity. The growth and progress of advanced RNA-directed technologies allow researchers to explore functions of genome “dark matter”. The greatest burden, however, is the tremendous and ambiguous amount of data generated during RNA-seq, which requires further interpretation. Moreover, lncRNA action is highly context-dependent, and the subcellular localization of RNA molecules seems to be fundamental. The dynamics of how the compartmentalization is achieved constitute another question. Plenty of studies have been carried out to clarify the role of lncRNAs in cancer. These require a more comprehensive approach encompassing the complex signaling networks related to lncRNAs. Determination of possible tumor-inducing and tissue-specific lncRNAs raise hopes for development of new targeted antineoplastic agents [95].

## 4. Heat Shock Proteins (HSPs)

Heat shock proteins (HSPs) are a heterogeneous group of conservative chaperoning proteins discovered by Ferruccio Ritossa in 1960 [96,97]. HSPs differ in their molecular weight (ranging from 10 to more than 100 kDa) [98]. Differences in molecular weight make it possible to divide of HSPs into several classes. According to their molecular weight, HSPs can be grouped into several families: HSP27 (HSPB), HSP60 (HSPD), HSP70 (HSPA), HSP90 (HSPC), and HSP110 (HSPH) [99]. HSPs have a broad range of enzymatic activities, mostly associated with proper protein folding under both normal and stress conditions. HSPs prevent misfolding of newly synthesized proteins and ensure their spatial structure and function [100]. Almost all HSPs (except HSP27) have ATP-ase activity. Despite the lack of ATP-ase activity, HSP27 may participate in protein refolding through recruitment of other chaperones such as HSP70/HSP40 [99].

The expression of HSPs is tightly coordinated by heat shock factors (HSFs) that bind to regulatory elements called heat shock elements (HSEs), located upstream in the HSP gene promoters [101]. Inactive HSFs are cytosolic, monomeric proteins hyperphosphorylated in signaling cascades. Phosphorylated HSFs translocate to the nucleus, where trimers are formed. These trimeric complexes bind to HSEs and promote HSP expression [102]. Several factors, like hyperthermia, ionizing radiation and DNA-damaging agents have been shown to influence HSP expression. Heat stress is a known inductor of mitochondrial ROS, and, thus, HSPs were speculated to be involved in ROS-induced DNA damage response [102]. Indeed Abe et al. showed that treatment of WISH cells with hydrogen peroxide or adriamycin resulted in HSP70 transition to the nucleus. This indicates that HSP70 might exhibit a protective role against DNA damage and somehow facilitate DNA repair [103]. The potential mechanisms by which HSP70 confers enhanced DNA repair properties is a result of oxidative damage diminishing antioxidant and anti- inflammatory properties of the protein, which boosts reduction of ROS-associated DNA damage [104]. The same HSP protein was later shown to play a significant role in DNA repair of doxorubicin or cisplatin-treated, heat-shocked peripheral blood mononuclear cells (PBMCs). Moreover, it was estimated that these repair enhancing properties of HSP70 may be attributed to the observed higher expression of two MMR proteins, hMLH1 and hMSH2 [105,106,107]. Other HSPs have also been shown to be implicated in MMR-mediated DNA repair [108,109]. For instance, HSP90 was shown to stabilize MSH2 protein in pemetrexed treated human lung cancer cell lines [110]. The intimate association of HSP90 and MLH1 was also observed by Fedier et al. [111].

HSP proteins were also shown to play a significant role in radioresistance [112]. SiRNA interference of HSPs, including HSP72 (HSPA1A), led to impaired BER glycosylase activity and enhanced sensitivity to ionizing radiation in leukemic cells [113]. Moreover, HSPs have been shown to be involved in BER repair directly through stimulation of key pathway components, such as endonuclease APE1 [114], XRCC1 [115] and Polβ [116,117]. Furthermore, HSP70 (HSPA1A) was shown to associate with PARP-1, involved in SSB repair [118]. Moreover, HSP27 (HSPB1) may participate in excision of DNA damage in an NER-dependent manner, and subsequent downregulation of chaperone protein leads to impaired efficacy of UVC-induced damage removal [119].

The observation that HSP72 overexpression protects cells from UVC and benzo[a]pyrene damage accumulation and the results from other related studies suggest enhanced damage repair through the NER pathway [120,121,122,123]. HSPs also participate in NHEJ and HR-mediated DSB repair [124]. Of particular importance, HSP90 has been identified as a master regulator of many DNA repair components, such as BRCA1/2, RAD51, CHK1, DNA-PKsc, MRN complex, FA proteins and others, as reviewed by Pannisi et al. and Sottile and Nadin [61,124].

Although HSPs do not participate in DNA repair directly, it was discovered that they may modulate the activity of other DDR components. Changes in HSP expression strongly influence the efficacy of DNA repair, and thus compromise an important target of DDR-directed anticancer therapies. Moreover, hyperthermia has been shown to improve the treatment efficacy of many commonly used anticancer agents, as reviewed by Takemoto [125] and Urano [126], and therefore should be further investigated as a synergistic or adjuvant therapy in cancer treatment [127,128,129]. The role of HSPs in the eukaryotic DNA damage response system is summarized in Table 1.

## 5. Circadian Clock

Circadian rhythm (clock) compromises a basic mechanism that regulates many aspects of metabolism, biochemistry and behavior of all organisms [141]. Molecular clock is composed of positive (BMAL1, CLOCK) and negative factors (CRY 1/2, PER 1/2/3) that regulate transcription based on network of feedback mechanisms between transcription and translation in the transcription–translational feedback loops (TTFL). The major loop consists of circadian locomotor output cycles protein kaput (CLOCK) and brain and muscle ARNT-like 1 (BMAL1) complexes that regulate expression of period (*PER1/2/3*) and cryptochrome (*CRY1/2*) genes [142,143]. CLOCK and BMAL are class VII HLH proteins that contain PAS domains [144]. They form heterodimers that bind to E-boxes (CACGTG) in the promoters of the *CRY* and *PER* genes to effectively enhance their transcription.

After translation, CRY and PER proteins accumulate in the cytoplasm. This is followed by their heterodimerization and translocation to nucleus, and subsequent inhibition of CLOCK–BMAL1-mediated transcription, after a time delay. As a result, a negative feedback loop is formed. The new cycle begins after CRY and PER proteins are degraded [145].

**Figure 3:**
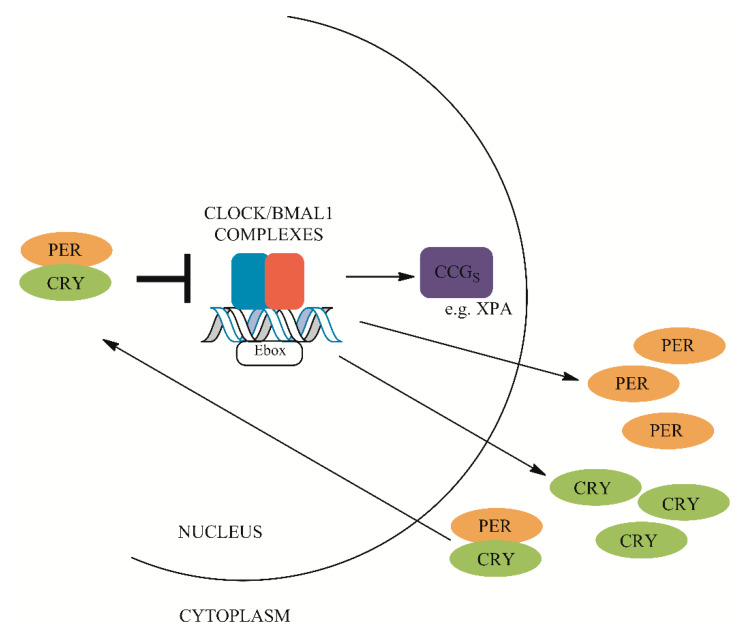
Transcription–translational feedback loop (TTFL) of the circadian clock. Circadian locomotor output cycles protein kaput (CLOCK) and brain and muscle ARNT-like 1 (BMAL1) complexes regulate the expression of period (*PER1/2/3*) and cryptochrome (*CRY1/2*) genes. After translation, CRY and PER proteins accumulate in the cytoplasm. This is followed by their heterodimerization and translocation to nucleus and subsequent inhibition of CLOCK–BMAL1-mediated transcription. CLOCK–BMAL1 complexes control expression of other clock-controlled genes (CCGs), such as the *XPA* gene.

Moreover, CLOCK–BMAL1 complexes control expression of other genes containing E-boxes, collectively known as clock-controlled genes (CCGs) (Figure 3) [146]. It has been estimated that expression of 2%–10% of mammalian genes is controlled by clock genes [147].

The central molecular clock, a pacemaker, is located in the anterior part of the hypothalamus called the suprachiasmatic nucleus (SCN). Peripheral clocks in individual tissues synchronize with each other and with the master clock located in the brain to effectively regulate intracellular processes such as DDR [146]. This synchronization is achieved through combination of both hormonal, humoral and neural inputs. Blue light is the strongest stimulus that entrains the central clock. Peripheral clocks, on the other hand, react to other stimuli [146,147,148]. The circadian clock has been reported to be involved in direct reversal of DNA damage through regulation of O6-methylguanine-DNA methyl transferase and alkylguanine DNA glycosylase.

Furthermore, the circadian clock regulates both ATR- and ATM-mediated DNA damage checkpoints involved in G1/S, G2/M cell cycle arrest and apoptosis. Several animal studies have revealed that activity and efficiency of some DNA repair systems may undergo circadian oscillations, and thus may function in a tissue-dependent manner. The basic example is NER. XPA protein involved in the damage recognition step during NER was shown to undergo circadian oscillations. Moreover, it was discovered that *XPA* genes contain two E-boxes in promotor regions, indicating that the level of XPA protein inside the cell may be regulated by the circadian clock components [149]. Furthermore, CLOCK and PER proteins have been shown to play a more direct role in DDR. CLOCK localized to the sites of DSBs after the DNA damage was found, and, therefore, one can speculate that it may somehow affect DSBR. PER2, on the other hand, directly associates and forms complexes with TP53 protein, preventing both ubiquitination of TP53 by MDM2 ligase and subsequent inhibition of the protein. Moreover, PER2 may be targeted by MDM2 ligase, indicating a more intrinsic relationship between PER proteins and TP53 [150]. Components of the molecular clock may be affected by other previously mentioned groups of DDR-related players. For example, SIRT1 directly deacetylates clock proteins affecting the gene expression of CCGs or directly affects the levels of acetylated histones in the promoters of the clock genes [151,152]. Furthermore, SIRT1 may act as a nutrient sensor that coordinates circadian clock with the energetic status of the cell [153]. SIRT1 is not the only sirtuin involved in circadian clock regulation. SIRT6 regulates the recruitment of CLOCK: BMAL1 complexes to the promoters of circadian genes and influences their expression [154].

## 6. Existing Crosstalks

As was previously mentioned, sirtuins have been shown to be involved in the circadian clock. A wide array of crosstalks between sirtuins, lncRNAs and HSPs have been recognized. For example, *Sirt1* antisense long noncoding RNA was shown to stabilize *Sirt1* mRNA, affecting SIRT1 protein expression in cardiomyocytes [155]. *MALAT1,* on the other hand, was shown to interact with FOXO1 and suppress SIRT1 transcription following high-glucose-induced damage in HK-2 cells [156]. In the same cell line, another lncRNA, *TUG1*, protected cells against lipopolysaccharide-induced inflammatory damage through regulation of miR-223 and SIRT1 expression [157]. Furthermore, SIRT1 promoted association of HSF1 with the *HSP70* gene promoter by maintaining HSF1 in a deacetylated state [158]. SIRT3 was demonstrated to target HSP10 for deacetylation and thus modulate mitochondrial protein folding following prolonged fasting conditions [159]. Moreover, the long noncoding RNA (lncRNA) *NEAT1* contains a heat shock element in the promoter region and is identified as the transcriptional target of HSF1. Moreover, *NEAT1* expression is controlled by HSF1, which binds to the heat shock element located in the promoter region of *NEAT1* lncRNA [160]. The relationships between non-coding RNAs and heat shock response in mammals have been extensively reviewed by Place and Noonan [161]. Cui et al. reported a case where lncRNA *HULC* increased the expression of CLOCK protein and downstream circadian oscillators. This may suggest the interdependence between lncRNAs and the circadian clock [162]. Mouse-based studies also revealed that some circadian lncRNAs had analogous circadian phase oscillations, the same as genes closely located in their proximity. These lncRNAs were shown to be mainly expressed from enhancer regions through BMAL-dependent transcription [163]. Similarly, HSF1 was found to undergo rhythmic circadian oscillations and regulate expression of HSPs at the onset of the circadian dark phase in rodents [164]. Furthermore, clock components such as BMAL1 were shown to act as important clients for HSP proteins in vitro [165]. Altogether, these findings suggest a sophisticated interplay between different classes of DNA-damage-related classes of molecules. However, crosstalks regarding DNA damage and repair remain to be elucidated.

## 7. Other Players

Other DDR-related proteins have recently been shown to play a significant role in DNA damage and repair. A basic site processing protein, HMCES, is one of the most recently discovered pivotal players in BER. Evolutionarily conserved HMCES forms DNA–protein crosslinks that protect abasic sites from turning into SSBs upon action of AP endonucleases and act to secure DNA from the consequences of error-prone DNA polymerase activity at stalled replication forks [166,167,168]. Recently, the same protein was shown to be involved in DSBR repair during class switch recombination in B cells. This further emphasizes the role of HMCES in genomic stability maintenance [169]. Another protein involved in BER, DNA2, was shown to promote the restart of arrested replication forks by working in concert with Werner syndrome ATP-dependent helicase (WRN) and Bloom syndrome protein (BLM). Furthermore, it was shown that DNA2 with other factors is involved in the resection step during HR. The extensive role of DNA2 helicase/nuclease in DNA repair was summarized elsewhere by Pawłowska et al. and Zheng et al. [170,171]. Stefanovie et al. established another important contributor to DSBR. A small, acidic protein called DSS was shown to play a crucial role in stimulation of RAD52 oligomer formation and consequent strand invasion during single-strand annealing (SSA) and break-induced replication (BIR) repair processes [172]. Moreover, new emerging functions of RAD52 in DNA repair have been proposed by Jalan et al. [173]. Effective recruitment of many DNA repair factors can be facilitated by PARPs. Despite extensive research carried on the role of PARPs in DNA repair, new functions of these enzymes are being discovered. Unquestionably, they play a prominent role in DSBR, SSBR and BER repair pathways and their activity and molecular clients are expanding [174].

## 8. Conclusions

Over the last decade, significant scientific progress in the understanding of DNA damage and repair has been made. Despite extensive knowledge about the core components of DNA repair pathways, new non-classical players in the area of eukaryotic DDR have been recognized. Sirtuins represent an important group of DDR regulatory proteins that affect both chromatin condensation status and repair efficacy. On the other hand, lnRNAs compromise a group of molecules with diverse functions. The versatility of lnRNAs in the control of DNA repair results from their capacity to regulate chromatin remodeling, allowing effective recruitment of repair components to the sites of damage, regulation of *TP53* on both transcriptional and translational level, and sponging of DDR-related miRNAs. Chaperoning of DDR-components further affects the complexity of repair processes and constitutes an interesting field of research. Moreover, given the number of genes regulated by the circadian clock, new targets in DDR will surely be explored in the future. Understanding of their individual contributions to genomic stability maintenance and comprehension of existing crosstalks seems to be crucial and may lead to development of novel treatment strategies for cancer, age-related diseases and more. Knowledge regarding non-classical repair pathways may comprise a path for the development of new anticancer agents and constitute a pool of potential molecular targets for targeted therapies.

## Figures and Tables

**Table 1:** The role of HSPs in eukaryotic DDR mechanisms. The key DNA repair components were provided with types of lesions repaired during DDR [2,100] with modifications.

DDR Mechanisms	Type of DNA Lesion	Key Components	HSP	Partner	Effect on DNA Repair	Reference
Direct DNA-lesion reversal	- O6 alkylguanine	O6-methylguanine methyltransferase (MGMT)	HSPC2 (Hsp90α), HSPC3 (Hsp90β)	MGMT	Not clear	[130]
Base excision repair (BER) and single-strand break repair (SSBR)	Chemically modified DNA bases (DNA adducts; oxidized bases; alkylated bases; single-strand breaks)	DNA glycosylases, APE1 endonuclease, DNA polymerases (β, δ, ε), flap endonuclease FEN1, ligase I or ligase III, XRCC1, PARP enzymes (PARP-1, PARP-2), DNA ends- modifying enzymes polynucleotide kinase (PNK), aprataxin (APTX), tyrosyl-DNA phosphodiesterase 1 (TDP1)	HSP70	APE1	Stimulation of DNA repair	[114]
				Polβ	Stimulation of DNA repair	[116,117]
			HSP90	XRCC1	Choice between DNA repair mechanism (polymerase-β-dependent or -independent)	[115]
			HSP70	PARP1, XRCC1	Stimulation of SSBR repair	[118]
Nucleotide excision repair (NER)	Lesions that significantly disrupt the DNA double-helix (massive DNA adducts; 6’-4’ photoproducts; cyclobutane pyrimidine dimers (CPDs))	XP proteins, RNA polymerase, XPC-HR23B DDB1/2	HSP27	Not identified	Stimulation of NER	[119]
			HSP70	XPA and XPG	Not identified	[123]
Mismatch repair (MMR)	- DNA mismatches - insertion/deletion loops	protein complexes (MSH2-MSH6, MSH2-MSH3 MLH1-PMS2 MLH1-PMS1, PLH1-MLH3), EXO1, polymerases δ and ε, PCNA, RFC, RPA, ligase I	HSP27/HSP70	MSH2/MLH1	Not identified	[107]
			HSP90	MSH2	Stabilization of the interacting partner	[110]
Trans-lesion synthesis (TLS)	- damaged bases that prevent replication fork progression	“Error-prone” DNA polymerases	HSP90	TLS polymerases	Promotes TLS activity in plants	[131]
Non-homologous end- joining (NHEJ)	- double-strand breaks (DSBs)	Ku 70/80, DNA-PKcs, XRCC4, XLF/cernunnos, ligase IV, Artemis nuclease, PNK, Aprataxin and polymerases μ and λ	HSP27	Ku80	Prevention of Ku80-DNA-PKcs interactions	[132]
			HSP90	DNA-PKcs	Activation and stabilization of DNA-PKcs for efficient repair	[133]
			HSP110	Ku70/Ku80	Recruitment of NHEJ proteins (Ku70/80, DNA-PKCS) for efficient repair	[134]
Homologous recombination (HR)	- double-strand breaks (DSBs) - inter- and intrastrand crosslinks (ICLs) - stalled replication forks - abortive topoisomerase II action	RAD51 and RAD51-related protein, RAD52, BRCA2, RPA, FEN1, DNA polymerases, MRN, CtIP, BRCA1	HSP90	BRCA2	RAD51 foci formation and effective DSB repair	[135]
				MRN	MRN/ATM/ATR complex stabilization	[136]
Fanconi anemia (FANC) pathway	- inter-strand DNA cross-links	FA-proteins	HSP90	FANCA	Stabilization of FANCA	[137]
ATR mediated DDR signaling	- single-strand breaks (SSBs)	RPA, ATRIP, RAD9-RAD1-HUS1 (911) complex, ATR, MRN, CtIP, TOPBP1, Claspin	HSP90	ATR	ATR is a direct client of HSP90, exact function remains to be elucidated	[138]
ATM mediated DDR signaling	- double-strand breaks (DSBs)	MDC1, 53BP1, RNF8	HSP27	ATM	Required for ATM-mediated DSBR repair upon radiation	[139]
		RNF168, BRCA1, ATM, MRN, CHK2	HSP90	ATM	Required for ATM/ATR mediated HR repair upon radiation and replicative stress	[140]

## Abbreviations

APE1	AP endonuclease 1
APTX	Aprataxin
ATM	Protein kinase ataxia–telangiectasia mutated
ATR	Protein kinase ataxia–telangiectasia and Rad3-related
ATRIP	ATR interacting protein
BER	Base excision repair
BIR	Break-induced replication
BLM	Bloom syndrome protein
BMAL1	Brain and muscle ARNT-like 1
BRCA1	Breast cancer type 1 susceptibility protein
CCG_S_	Clock-controlled genes
CDK	Cyclin-dependent kinase
CHK1/2	Serine/threonine-protein kinase Chk1/2
CLOCK	Circadian locomotor output cycles protein kaput
CPD	Cyclobutane pyrimidine dimers
CRY1/2	Cryptochrome-1
CtIP	CtBP-interacting protein
DDB1/2	DNA damage-binding protein
DDR	DNA damage Response
DNA2	DNA replication ATP-dependent helicase/nuclease DNA2
DNA-PK_CS_	DNA-dependent protein kinase, catalytic subunit
DSB	Double-strand break
DSS1	DSS1 protein
EXO1	Exonuclease 1
FANC	Fanconi anaemia pathway
FEN1	Flap endonuclease 1
HLH	Helix–loop–helix motif
HMCES	5-Hydroxymethylcytosine binding, ES-cell-specific
hnRNP-K	Ribonucleoprotein-K
HR	Homologous recombination
HR-23B	UV excision repair protein RAD23 homolog B
HSF	Heat shock factor
HSP	Heat shock protein
ICLs	Inter-/intrastrand crosslinks
lncRNA	Long-noncoding RNA
MDC1	Mediator of DNA damage checkpoint protein 1
MGMT	O6-methylguanine methyltransferase
MLH1	MutL homolog 1
MMR	Mismatch repair
MMS	Methyl-methanesulfonate
MRE11	MRE11 homolog, double-strand break repair nuclease
MRN	MRE11, RAD50 and NBS1 complex
MSH2/3/6	MutS homolog 2/3/6
MYH	MutY homolog
NBS1	Nibrin
NER	Nucleotide excision repair
NHEJ	Non-homologous end joining
OGG1	8-oxoguanine DNA glycosylase
PARP	Poly(ADP-ribose) polymerase
PAS	Per-Arnt-Sim domain
PBMCs	Peripheral blood mononuclear cells
PCNA	Proliferating cell nuclear antigen
PER1/2/3	Period circadian protein homolog 1
PMS	Mismatch repair endonuclease PMS2
PNK	Polynucleotide kinase
RAD51	RAD41 recombinase
RFC	Replication factor C
RNF168	Ring finger protein 168
ROS	Reactive oxygen species
RPA	Replication protein A
SCN	Suprachiasmatic nucleus
SIRT	Sirtuin
SNF2h	Sucrose nonfermenting-like 5
SSA	Single-strand annealing
SSB	Single-strand break
SSBR	Single-strand break repair
TDG	Thymine DNA glycosylase
TDP1	Tyrosyl-DNA phosphodiesterase 1
TLS	Trans-lesion synthesis
TOPBP1	DNA topoisomerase 2-binding protein 1
TP53	Cellular tumor antigen p53
TP53BP1	Tumor suppressor p53-binding protein 1
TTFL	Transcription–translational feedback loop
WRN	Werner syndrome ATP-dependent helicase
XP	Xeroderma pigmentosum protein
XRCC1	X-ray repair cross-complementing protein 1
γH2AX	Phosphorylated histone protein H2AX
